# A real-world retrospective, observational study of first-line pembrolizumab plus chemotherapy for metastatic non-squamous non-small cell lung cancer with PD-L1 tumor proportion score < 50% (PEMBROREAL)

**DOI:** 10.3389/fonc.2024.1351995

**Published:** 2024-03-27

**Authors:** Alessandro Cafaro, Flavia Foca, Oriana Nanni, Marco Chiumente, Marina Coppola, Paolo Baldo, Sabrina Orzetti, Fiorenza Enrico, Vito Ladisa, Rosa Lerose, Patrizia Nardulli, Piera Maiolino, Federica Gradellini, Anna Rita Gasbarro, Gisella Carrucciu, Riccardo Provasi, Paola Cristina Cappelletto, Alessandra Pasqualini, Stefano Vecchia, Marianna Veraldi, Adele Emanuela De Francesco, Lucio Crinò, Angelo Delmonte, Carla Masini

**Affiliations:** ^1^ Pharmacy Unit, IRCCS Istituto Romagnolo per lo Studio dei Tumori (IRST) “Dino Amadori”, Meldola, Italy; ^2^ Unit of Biostatistics and Clinical Trials, IRCCS Istituto Romagnolo per lo Studio dei Tumori (IRST) “Dino Amadori”, Meldola, Italy; ^3^ Scientific Direction, Società Italiana di Farmacia Clinica e Terapia (SIFaCT), Turin, Italy; ^4^ Pharmacy Unit, IRCCS Istituto Oncologico Veneto (IOV), Padova, Italy; ^5^ Pharmacy Unit, CRO Aviano IRCCS, National Cancer Institute, Aviano, Italy; ^6^ Hospital Pharmacy, Candiolo Cancer Institute, FPO-IRCCS, Candiolo, Turin, Italy; ^7^ Hospital Pharmacy, IRCCS National Cancer Institute Foundation, Milan, Italy; ^8^ Hospital Pharmacy, IRCCS-CROB Referral Cancer Center of Basilicata, Rionero in Vulture, PZ, Italy; ^9^ Pharmacy Unit, National Cancer Research Center Istituto Tumori “Giovanni Paolo II”, Bari, Italy; ^10^ Pharmacy Unit, Istituto Nazionale Tumori “Fondazione G. Pascale”, IRCCS, Naples, Italy; ^11^ Pharmacy Unit, Azienda USL-IRCCS di Reggio Emilia, Reggio Emilia, Italy; ^12^ Pharmacy Unit, University Hospital Policlinico, Bari, Italy; ^13^ Pharmacy Unit, Azienda Ospedaliera Brotzu, Cagliari, Italy; ^14^ Pharmacy Unit, Azienda Sanitaria Universitaria Giuliano Isontina, Trieste, Italy; ^15^ Hospital Pharmacy, Hospital of Bolzano (SABES-ASDAA), Bolzano, Italy; ^16^ Pharmacy Unit, S.Chiara Hospital, Trento, Italy; ^17^ Pharmacy Unit, Hospital Guglielmo da Saliceto, Piacenza, Italy; ^18^ Protesic and Pharmaceutical Assistance sector n. 3, Department of Health Protection and Health Service Calabria Region, Catanzaro, Italy; ^19^ Pharmacy Unit, Mater Domini Hospital, Catanzaro, Italy; ^20^ Thoracic Oncology Unit, IRCCS Istituto Romagnolo per lo Studio dei Tumori (IRST) “Dino Amadori”, Meldola, Italy

**Keywords:** pembrolizumab, non-small cell lung cancer, real-world data, immunotherapy, observational study

## Abstract

**Introduction:**

The phase III Keynote-189 trial established a first-line treatment combining pembrolizumab with pemetrexed and platinum as a standard treatment for patients with stage IV non-small cell lung cancer (NSCLC) without known *EGFR* and *ALK* driver mutations and independent of programmed cell death ligand 1 (PD-L1) expression. However, in Italy, eligibility for the National Health Service payment program is limited to patients with PD-L1 <50%. The PEMBROREAL study assesses the real-world effectiveness and safety of pembrolizumab in patients eligible for the National Health Service payment program.

**Methods:**

PEMBROREAL is a retrospective, observational study on patients with NSCLC who started pembrolizumab combined with pemetrexed and platinum within the reimbursability time window, considered as December 2019 to December 2020. The primary endpoints were to assess progression-free survival (PFS) and overall survival (OS; using the Kaplan–Meier method), response to therapy, and tolerability.

**Results:**

Until February 2022, 279 patients (median follow-up: 19.7 months) have been observed. The median PFS was 8.0 months (95% confidence interval: 6.5–9.2). OS was not reached, but we can estimate a 12- to 24-month survival rate for the combined treatment: 66.1% and 52.5%, respectively. PD-L1 expression and Eastern Cooperative Group (ECOG) Performance Status were both associated with PFS and OS. Overall, only 44.4% of patients reported an adverse event, whereas toxicity led to a 5.4% discontinuation rate.

**Conclusion:**

The results of the PEMBROREAL study have shown that the combined treatment of pembrolizumab with pemetrexed and platinum is effective for metastatic non-squamous NSCLC, even for patients with PD-L1 levels below 50%, despite the differences in patient demographics and pathological features compared to the Keynote-189 study. The adverse events reported during the study were more typical of chemotherapy treatment rather than immunotherapy, and physicians were able to manage them easily.

## 1 Introduction

Randomized clinical trials (RCTs) are undoubtedly the best way to highlight the benefits of a novel technology or intervention in healthcare under ideal circumstances and, therefore, avoid confounding factors that could either enhance or cover the effects arising from its implementation. The results from an RCT are very important considering that they can dramatically change the standard practices and could improve therapeutic offers for patients (1).

Not less important is verifying whether the RCT results can improve outcomes in real-world practice, outside the ideal context of a clinical trial, in which patients are selected with rigorous inclusion and exclusion criteria and procedures and timing are strictly defined (2–6).

A.L. Cochrane defines effectiveness as the measure of whether an intervention provides more benefits than drawbacks when applied in regular healthcare settings (7). Therefore, the most reliable method to assess the effectiveness of novel technology is to conduct a randomized pragmatic study in regular clinical practice. This study would compare the outcomes of two treatments used to address the same medical condition (8–11).

However, it is not ethical to deny a group of patients a treatment that has been proven effective through a randomized controlled trial. Additionally, scientific advancements and news spread rapidly across the globe, making information about the latest approved treatments easily accessible to patients and their caregivers.

The only way to assess treatment effectiveness is through real-world studies even though they are not designed to evaluate comparative effectiveness and suffer from bias and confounding factors (12). Real-world studies are nevertheless important for the following:

1) patients firstly who are not interested in how a particular therapy can benefit the general population but are only concerned about obtainable results for conditions like theirs;
2) clinicians who can determine which patients will obtain the most benefits from a particular treatment and those who will obtain fewer benefits; and
3) healthcare administrators who are interested in understanding if a new technology is cost-effective or not (13, 14).

Immunotherapy has become the most impactful technology in the field of oncology over the past decade (15). It has redefined treatment guidelines for most forms of cancer, especially for drugs that are effective on the programmed cell death protein 1/programmed cell death ligand 1 (PD-1/PD-L1) signaling pathway, since nivolumab has been approved by the National Health System for the treatment of metastatic melanoma and second-line therapy of non-small cell lung cancer (NSCLC) (16, 17). Soon after, other drugs like pembrolizumab, atezolizumab, avelumab, durvalumab, cemiplimab, and dostarlimab became available for many other indications beyond the first two registered (18). These drugs have made it possible to treat cancers like NSCLC that a decade ago had a poor prognosis but now have a better life expectancy (19). Pembrolizumab was the first drug active on the PD-1/PD-L1 axis available for the first-line treatment of metastatic NSCLC. Firstly, the availability was limited to the treatment as monotherapy for patients with a tumor proportional score (TPS) of PD-L1 ≥ 50% without the driver mutations epidermal growth factor receptor (*EGFR*) and anaplastic lymphoma kinase (*ALK*) (20). Subsequently, pembrolizumab in combination with pemetrexed and platinum became available and reimbursed for patients with nonsquamous NSCLC with TPS PD-L1 <50%. This could be possible thanks to the outcome of the Keynote-189 trial, in which pemetrexed and platinum combined with pembrolizumab achieved better results than that combined with placebo in OS [median: 19.4 months vs. 11.3 months; hazard ratio (HR): 0.60; 95% confidence interval (CI): 0.50–0.72] and PFS (median 9.0 months vs. 4.9 months; HR: 0.50; 95% CI: 0.42–0.60) both in the intention-to-treat population and in subgroups stratified for TPS of PD-L1 (21).

The Italian Medicinal Agency (AIFA) reimburses patients with central nervous system metastasis, whereas they would have been exclusion criteria for the Keynote-189 study unless they were previously treated for brain metastases, were clinically stable for at least 2 weeks, showed no evidence of new or enlarging brain metastases, and had been off steroids 3 days before dosing with pembrolizumab. In standard clinical practice, patients were treated only if they were not symptomatic for central nervous system metastasis, although with less caution regarding the stability period post-radiation treatment and the steroid washout period.

Moreover, patients with Eastern Cooperative Group Performance Status (ECOG PS) 2, who would be excluded from Keynote-189, are eligible for National Health Service payments.

On the other hand, although two meta-analyses proved that chemo-immunotherapy was more effective than immunotherapy alone (22, 23), only patients with PD-L1 < 50% met the reimbursement criteria for the combined pembrolizumab, pemetrexed, and platinum treatment. In this subgroup, Keynote-189 demonstrated the superiority of immunotherapy as compared to placebo when combined with pemetrexed and platinum. The results were based on a post-hoc analysis with a sample not calibrated to reject the null hypothesis with a predefined alpha error. Moreover, as always, the exclusion criteria for patients with a life expectancy of less than 3 months present in the clinical trial protocol are not applicable in real-world practice. Key features of patients treated in real-life settings and the registrative clinical trials are summarized in **Supplementary Table 1**.

To evaluate the real benefit of pembrolizumab in a real-world context, considering the Italian Medical Agency’s reimbursability criteria, we designed an observational retrospective cohort study (PEMBROREAL) in 16 Italian oncology institutions to assess the effectiveness and safety of pembrolizumab in combination with pemetrexed and platinum used for first-line therapy of NSCLC. The study aims to describe the benefit not only for the general population but also for specific subgroups of interest.

## 2 Materials and methods

### 2.1 Study design

PEMBROREAL is an Italian, retrospective, multicenter study of a cohort of patients who received at least one administration of pembrolizumab plus chemotherapy for previously untreated non-squamous metastatic NSCLC without *EGFR* and *ALK* driver mutations. The study reviews medical records for a subset of adult patients with non-squamous NSCLC consecutively treated with pembrolizumab combined with pemetrexed and platinum (carboplatin or cisplatin) at one of the 16 study centers, according to reimbursability criteria established by the Italian Medicinal Agency. We planned chart extractions at two pre-specified dates for each participant: the first in January 2021, and the last in February 2022. The first data extraction was done to ensure that the participating centers met a high standard of data collection. In contrast with the inclusion and exclusion criteria of the registrative Keynote-189 trials, the reimbursability rules in Italy approved the pembrolizumab treatment for patients with ECOG PS 2, with active central nervous system metastasis, and with a life expectancy of less than 3 months. However, only patients with TPS of PD-L1 < 50% could be treated with the combination of pembrolizumab, pemetrexed, and platinum. To be enrolled, patients had to start the combination treatment pembrolizumab, pemetrexed, and platinum within the allocated reimbursability time window from December 2019 to the end of December 2020, and have provided informed consent for data to be retrieved from their medical records. Patients who were unreachable, despite every reasonable effort, or who died were included in the study according to national law. Patients being treated with pembrolizumab inside an interventional clinical trial, in a compassionate-use program or off-label, were excluded. The study protocol was approved by the Ethics Committee of each participating institution and conducted following the 1964 Declaration of Helsinki guidelines and its later amendments.

### 2.2 Assessments

The study aimed to assess the effectiveness, safety, and activity of the combined use of pembrolizumab with pemetrexed and platinum for non-squamous NSCLC, based on the criteria for reimbursement set by the Italian Medicinal Agency. The goal was to determine if the results obtained in the clinical trial reflect the outcomes observed in real-world practice for patients receiving this combination treatment. The primary endpoints were (1) real-world progression-free survival [RwPFS—measured by the index date, intended as the first pembrolizumab administration, to the date of progression assessed by treating physicians or death (if no progression), or the end of follow-up whichever comes first]; (2) overall survival (OS—measured from the index date to death, or the end of follow-up whichever comes first); (3) overall response rate (ORR) was defined as the proportion between patients with complete or partial response and the overall patients; similarly, disease control rate (DCR) was calculated as the rate between patients with a complete or partial response, stable disease on overall patients; (4) duration of response [DoR—measured from the date of first response documented in medical records until disease progression, or death (if no progression)]; patients without evidence of progression were censored at the end of follow-up; (5) incidence and management of adverse events (AEs) as identified by the treating healthcare practitioner; and (6) grade of AE (the reporter assigned grade as defined by the Common Terminology Criteria for Adverse Event, version 4). Given the real-world nature of PEMBROREAL, progression and response to therapy could be determined either by the physician’s assessments or according to the Response Evaluation Criteria in Solid Tumours (RECIST) version 1.1, depending on local practice.

Key secondary endpoints included the following: (1) RwPFS and OS for subgroups of interest; and (2) demographic, patients, and disease characteristics and details of administered therapy (for example, the platinum salt selected for combination therapy).

### 2.3 Statistical analyses

A formal calculation of sample size was not done. Descriptive statistical analyses were performed. Categorical variables were presented as frequency tables (absolute and relative), whereas for continuous variables, descriptive statistics as median with minimum and maximum values were shown. The RwPFS and OS data were censored for patients lost to follow-up (i.e., still alive as of their last visit or contact before the database cutoff). Median and landmark rates were calculated by the Kaplan–Meier method and the corresponding 95% CI was calculated by Greenwood’s method.

The analyses were based on the entire population of subjects enrolled in the study; subgroup analyses (i.e., stratified by disease, therapy, or other demographic or prognostic variables) were performed following the indications from the investigators of the participating centers. Log-rank test was performed to assess the potential prognostic role of factors identified by the investigators. A multivariable Cox regression model was performed to assess independent prognostic factors. Proportion hazard assumptions were checked through Schoenfeld residuals. A *p*-value <0.05 was considered statistically significant. Statistical analysis was carried out using STATA/MP 15.0 for Windows (StataCorp LP, College Station, TX, USA).

## 3 Results

### 3.1 Patient and hospital site characteristics

Until 28 February 2022 (the end date of the second chart extraction), 279 eligible patients had been identified. Patients were enrolled across 16 hospital sites, including nine Institutes for Cancer Treatment and Research, four university general hospitals, and three multi-specialty general hospitals. For most of the hospitals participating in the study (56%), the only field of care and research were oncology and onco-hematology. Eighty-one percent of the participating institutions (13/16) had a recognized role in cancer research. The median follow-up duration in the full analysis set was 19.7 months (range: 0.1 to 26.1); 22 patients (7.9%) were lost to follow-up. Two additional patients were potentially eligible for PEMBROREAL but were not enrolled because they had been treated before December 2019 and no longer fulfilled the reimbursement criteria set by the Italian Medicinal Agency.

### 3.2 Demographics and disease characteristics

The median age of the patients in the full analysis set was 65.5 years (minimum–maximum: 34.2–79.3) at first pembrolizumab administration; only 7.9% were aged above 75 years. Most of the patients were men (67.0%) and had a PS of 0 or 1 (94.1%) at first pembrolizumab administration. All the patients had a non-squamous histologic tumor type [97.9% adenocarcinoma and 2.1% NOS (not otherwise specified) histology] according to the indication approved for the combination therapy of pembrolizumab with pemetrexed and platinum. TPSs of PD-L1 were immunohistochemically evaluated by a validated 22C3 IHC laboratory test for all patients. A total of 123 (44.1%) patients had PD-L1% expression < 1% and 156 (55.9%) had an expression greater than or equal to 1% but less than 50%. Forty-eight patients (17.6%) were diagnosed with brain metastasis. For six patients included in the study, the presence or absence of brain metastasis was not documented. Smoker status was known for 141 (50.5%) patients, most of whom are current or former smokers (76.6%). Following the reimbursability criteria set by the Italian Medicinal Agency, *EGFR* and *ALK* mutations were tested using Next-Generation Sequencing (NGS) and immunohistochemical validated Ventana *ALK* (D5F3) and Rabbit Monoclonal, respectively, which resulted negative for 100.0% for all patients (**Table 1**). Other driver-gene mutations, (e.g., KRAS, BRAF, RET, and ROS), which might have affected the OS and PFS, were evaluated at the discretion of the participating centers. For this reason, our study did not collect data on the presence or absence of other driver mutations.

**Table 1:** Patient demographics and disease characteristics.

Characteristics	Number of patients *n* = 279 (%)
Age (continuous)	
Median (range)	65.5 (34.2-79.3)
Age (categorical)	
<75	257 (92.1)
≥75	22 (7.9)
Sex	
Male	187 (67.0)
Female	92 (33.0)
Histology	
Adenocarcinoma	273 (97.9)
NOS carcinoma	6 (2.1)
Large cell lung cancer	0 (0.0)
Adenosquamous	0 (0.0)
Squamous	0 (0.0)
Unknown—N/D	0
EGFR status known	
Yes	279 (100.0)
No	0 (0.0)
EGFR mutation	
Negative	279 (100.0)
ALK status known	
Yes	279 (100.0)
No	0 (0.0)
ALK status known	
Negative	279 (100.0)
PDL1	
<1%	123 (44.1)
1%–49%	156 (55.9)
≥50%	0
PS ECOG	
0	107 (39.5)
1	148 (54.6)
2	16 (5.9)
Unknown	8
Smoking history	
Ex-smoker	59 (41.8)
Smoker	49 (34.8)
Not smoker	33 (23.4)
Unknown	138
Presence of brain metastases	
Yes	48 (17.6)
No	225 (82.4)
Unknown	6

Percentages reported in the table were calculated using the number of patients with available data (for each variable).

### 3.3 Characteristics of pembrolizumab treatment

At the time of database cutoff, the median total treatment duration (including the duration of dose interruptions) was 176 days. Overall, 30.8% of patients received pembrolizumab for a total duration of more than 12 months. Patients received a median of 8.0 pembrolizumab infusions (range: 1–35). No participant received more than 35 cycles (equivalent to 2 years of treatment) due to the Keynote-189 study and reimbursement criteria. Overall, approximately 9.0% of patients experienced a temporary treatment interruption for more than 3 weeks for any cause. Pembrolizumab could be administered in combination with pemetrexed and either carboplatin or cisplatin. Most of the patients received carboplatin (80.3%) as a combination therapy with pemetrexed and pembrolizumab.

### 3.4 Reason for discontinuing pembrolizumab

Owing to the limited follow-up period, none of the patients in the study completed the maximum number of 35 treatment cycles. The most common reasons for treatment discontinuation were disease progression (55.2%) and death (7.5%). Only 5.4% of patients discontinued treatment with pembrolizumab due to unacceptable toxicity. At the time of data cutoff, 20.8% of patients continued to receive treatment with pembrolizumab (**Supplementary Table 2**).

### 3.5 Analysis of real-world PFS

**Figure 1:**
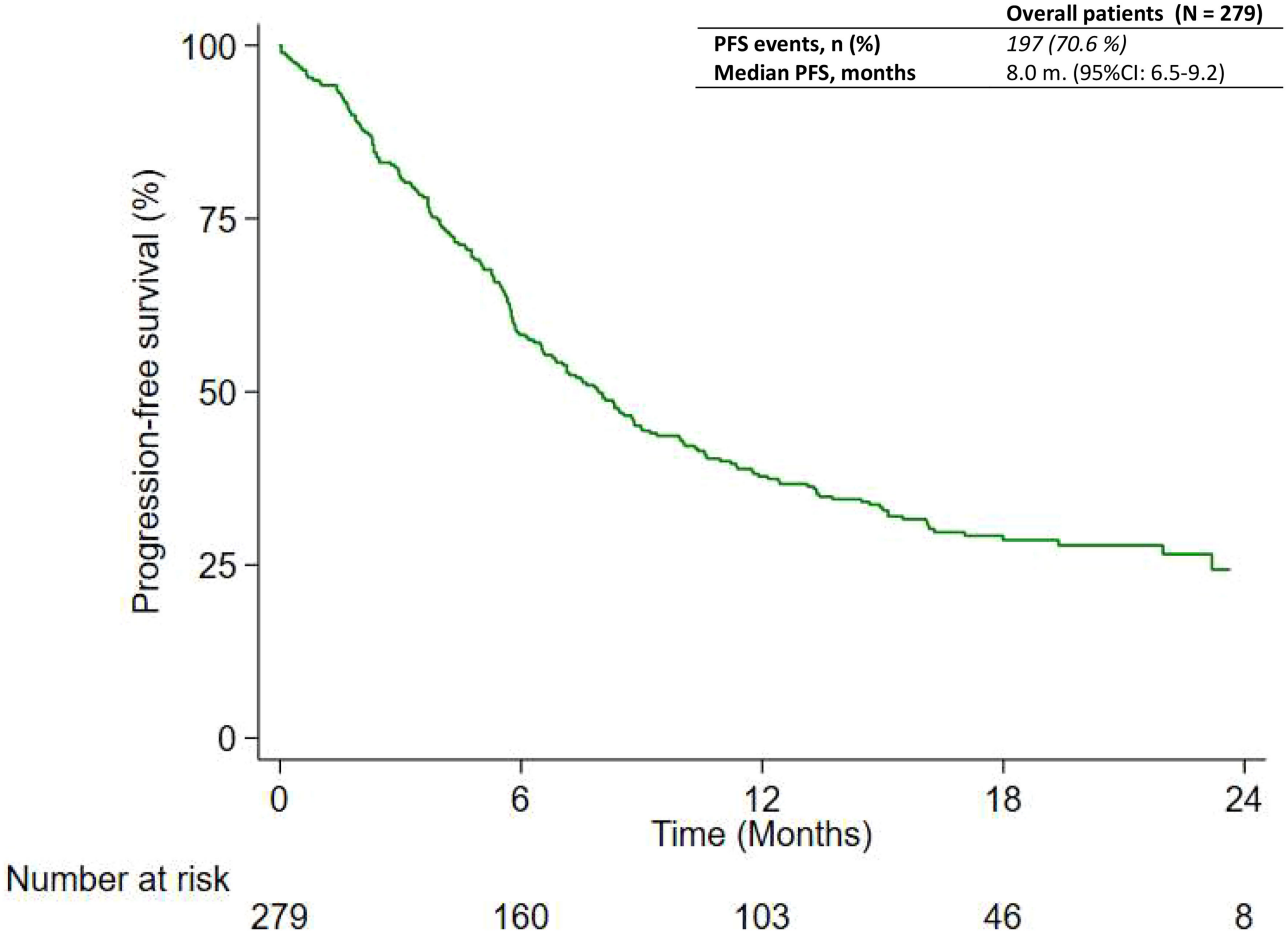
Progression-free survival of patients shown as Kaplan-Meier distribution.

At the time of data cutoff, 197 out of 279 patients (70.6%) had experienced disease progression or died without documentation of disease progression. Progression was determined per RECIST or clinical assessment. A radiological evaluation was made in local clinical practice (usually every 3 months, but this was not always possible for logistic or clinical reasons). The median RwPFS was 8.0 months (95% CI: 6.5–9.2) as reported in **Figure 1**. Subgroup analyses were performed to evaluate possible associations between RwPFS and prognostic factors of interest. As found in **Table 2**, RwPFS was significantly longer among patients with PD-L1 greater than or equal to 1% (median: 9.9 months; 95% CI: 7.6–11.8) versus patients with PD-L1 less than 1% (median: 5.8 months; 95% CI: 5.3–8.0; *p*-value: 0.009) (**Supplementary Figure 1A**), and among patients with ECOG PS 0 (median: 11.3 months; 95% CI: 8.3–17.0) versus ECOG PS 1 or 2 (median: 6.5 months; 95% CI: 5.6–8.1; *p*-value: 0.013) (**Supplementary Figure 1B**). Meanwhile, RwPFS was not different among patients aged less than 75 (median: 7.9 months; 95% CI: 6.5–9.2) versus those aged greater than or equal to 75 years (median: 8.3 months; 95% CI: 5.1–16.2; *p*-value: 0.853) but also among patients who received carboplatin (median: 7.1 months; 95% CI: 6.2–8.8) or cisplatin (median: 10.4 months; 95% CI: 7.4–14.5; *p*-value: 0.247). No difference was also found among current or former smokers (median: 8.6 months; 95% CI: 6.5–13.3) versus never smokers (median: 7.5 months; 95% CI: 5.7–11.9; *p*-value: 0.288), and among patients with brain metastasis (median: 8.0 months; 95% CI: 5.3–15.0) and those without (median: 7.9 months; 95% CI: 6.5–9.2; *p*-value: 0.490). The patient’s sex also seems to be unrelated to RwPFS and was similar between male (median: 7.9; 95% CI: 5.9–10.0) and female patients (median: 8.0 months; 95% CI: 6.1–10.6; *p*-value: 0.470). The multivariable analysis in **Supplementary Table 5** confirms that patients with PD-L1 between 1% and 49% had a lower risk of progression (HR: 0.69; 95% CI: 0.52–0.92) compared to patients with PD-L1 <1%. Patients with ECOG PS 1–2 had a higher risk of progression compared to patients with ECOG PS 0, with an HR equal to 3.97 (95% CI: 1.86–8.48) as the main effect; in addition, a time-dependent effect was found to decrease over time (HR: 0.56; 95% CI: 0.38–0.82).

**Table 2:** Univariable analysis of progression-free survival.

	Number of patients	Number of events	Median PFS (95%CI)	*p*-value (log-rank test)
All cases	279	197	8.0 (6.5–9.2)	–
Age				
<75 years	257	181	7.9 (6.5–9.2)	0.853
≥75 years	22	16	8.3 (5.1–16.2)	
Sex				
Male	187	135	7.9 (5.9–10.0)	0.470
Female	92	62	8.0 (6.1–10.6)	
PDL1				
<1%	123	95	5.8 (5.3–8.0)	0.009
1%–49%	156	102	9.9 (7.6–11.8)	
Platinum salts				
Cisplatin	55	37	10.4 (7.9–14.5)	0.247
Carboplatin	224	160	7.1 (6.0–8.8)	
PS ECOG				
0	107	66	11.3 (8.3–17.0)	0.013
1–2	164	124	6.5 (5.6–8.1)	
Smoking history				
Never smoker	33	26	7.5 (5.7–11.9)	0.288
Smoker or former smoker	108	70	8.6 (6.5–13.3)	
Presence of brain metastases				
Yes	48	32	8.0 (5.3–15.0)	0.490
No	225	160	7.9 (6.5–9.2)	

### 3.6 Analysis of real-world OS

**Figure 2:**
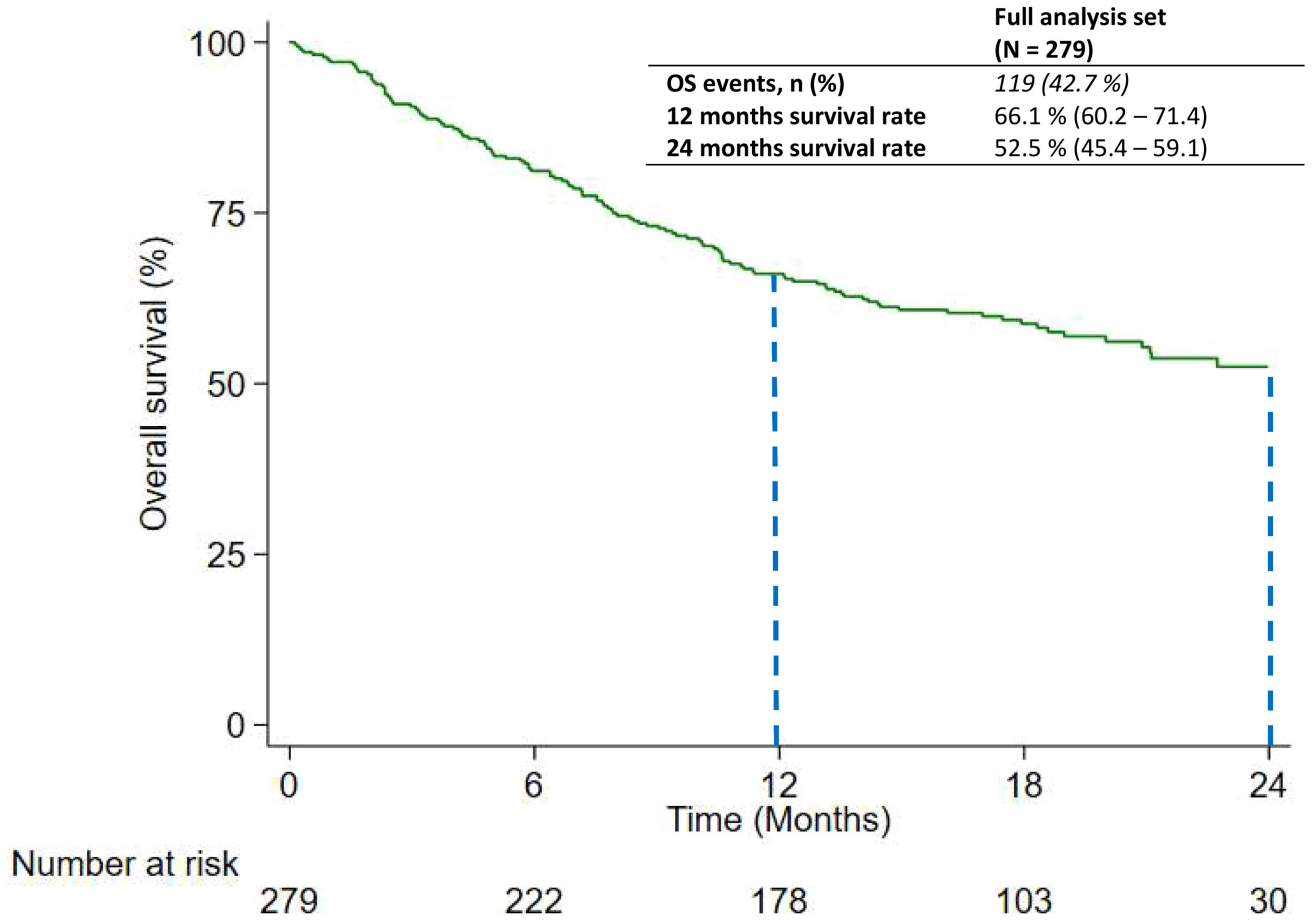
Overall Survival of patients shown as Kaplan – Meier distribution. Dashed lines represent 12- and 24-month landmark analyses.

At the time of the data cutoff, 119 out of 279 (42.7%) patients died. The median OS was not reached in the overall case series and 66.1% (95% CI: 60.2–71.4) of patients were estimated to be alive at 12 months, and 52.5% (95% CI: 45.4–59.1) were estimated to be alive at 24 months (**Figure 2**). The real-world OS (RwOS) subgroup was analyzed to evaluate any possible associations with prognostic factors. As found in **Table 3**, 12-month and 24-month survival rate were greater for patients with PD-L1 greater than or equal to 1% (71.8%; 95% CI: 63.9–78.2, and 59.4%; 95% CI: 50.1–67.5 at 12 and 24 months, respectively) versus PD-L1 less than 1% (59.0%; 95% CI: 49.8–67.2, and 43.2%; 95% CI: 32.3–53.6 at 12 and 24 months, respectively) (**Supplementary Figure 2A**). ECOG PS was also a prognostic factor related to survival considering that patients with ECOG PS 0 had a 77.2% (95% CI: 67.9–84.1) and 57.7% (95% CI: 41.9–70.7) survival rate at 12 and 24 months, respectively, versus 59.9% (95% CI: 51.9–67.0) and 49.6% (95% CI: 41.1–57.5) of patients with ECOG PS 1–2 (**Supplementary Figure 2B**). Female patients had a slightly higher survival rate than male patients, but the difference was not significant. All other prognostic factors taken into account in this study did not appear to have an effect on survival (age, carboplatin vs. cisplatin concomitant therapy, never smokers versus current or former smokers, and presence of brain metastasis). The multivariable analysis is shown in **Supplementary Table 6**: patients with PD-L1 between 1% and 49% had a lower risk of death (HR: 0.62; 95% CI: 0.43–0.90) compared to patients with PD-L1 <1%. Patients with ECOG PS 1–2 had a higher risk of death compared to patients with ECOG PS 0, with an HR equal to 1.63 (95% CI: 1.09–2.43); no time-dependent effect was found.

**Table 3:** Univariable analysis of overall survival.

	Number of patients	Number of events	12-month survival (95%CI)	24-month survival (95%CI)	*p*-value (log-rank test)
**All cases**	279	119	66.1 (60.2–71.4)	52.5 (45.4–59.1)	–
Age					
<75 years	257	108	66.3 (60.1–71.8)	53.2 (46.0–60.1)	0.691
≥75 years	22	11	63.6 (40.3–79.9)	0	
Sex					
Male	187	86	63.7 (56.3–70.1)	50.4 (42.4–57.9)	0.087
Female	92	33	71.1 (60.6–79.3)	57.1 (43.3–68.7)	
PDL1					
<1%	123	62	59.0 (49.8–67.2)	43.2 (32.3–53.6)	0.008
1%–49%	156	57	71.8 (63.9–78.2)	59.4 (50.1–67.5)	
Platinum salts					
Cisplatin	55	26	65.3 (51.1–76.3)	42.5 (24.9–58.9)	0.533
Carboplatin	224	93	66.3 (59.8–72.1)	54.8 (47.4–61.7)	
PS ECOG					
0	107	35	77.2 (67.9–84.1)	57.7 (41.9–70.7)	0.015
1–2	164	78	59.9 (51.9–67.0)	49.6 (41.1–57.5)	
Smoking history					
Never smoker	33	11	81.0 (62.5–91.0)	51.3 (25.5–72.2)	0.277
Smoker or former smoker	108	47	66.4 (56.6–74.4)	51.5 (40.2–61.7)	
Presence of brain metastases					
Yes	48	21	62.4 (47.2–74.4)	52.2 (34.6–67.2)	0.740
No	225	94	67.4 (60.8–73.2)	53.6 (45.9–60.6)	

### 3.7 Analysis of treatment response

Response to treatment was reported in 174 out of 279 patients (62.4%). Overall, 58 patients (20.8%) had a partial (19.0%) or complete response rate (1.8%) as the best response to treatment (**Supplementary Table 3**). For 19.4% of patients, the disease had progressed at the first evaluation. The DCR of the included patients was 79.2%. Between patients with a documented complete or partial response, the median DoR was 14.4 months (95% CI: 11.1–not estimable).

### 3.8 Safety

Out of 279 eligible patients, 124 (44.4%) reported at least one AE. Two types of AE were the most common: blood and lymphatic system disorders and gastrointestinal diseases were reported by 43.0% of patients who had received at least one administration of pembrolizumab combined with pemetrexed and platinum. The other AEs were related to skin and subcutaneous diseases (21.5%), general disorders and administration site conditions (36.6%), and an alteration of diagnostic exams (25.1%). A total of 27 patients reported grade 3–4 AEs, of which 11 were blood and lymphatic system disorders, 6 were gastrointestinal diseases, and 5 were general disorders and administration site conditions (**Table 4**). Most of the AEs reported did not require any management measure such as drug administration suspension, reduction, definitive interruption, hospitalization, or specific pharmacologic treatment. Among the patients who experienced an AE that required specific measures, 65 (23.3%) cases were managed with a specific pharmacologic treatment, 10.4% with a dose reduction of one of the drugs or all the drugs used in association with pembrolizumab, and 8.7% with the interruption of one or more drug used for cancer treatment (**Supplementary Table 4**). Only 15 (5.4%) patients definitively discontinued treatment due to toxicity.

**Table 4:** Adverse reactions reported.

Toxicities	Number of patients *n* = 279 (%)	
At least one adverse reaction	124 (44.4%)	
	Any grade	Grade 3–4
Infections and infestations	8 (2.9)	0
Blood and lymphatic system disorders	120 (43.0)	11 (7.8)
Immune system disorders	0 (0.0)	0
Endocrine disorders	7 (2.5)	0
Metabolism and nutrition disorders	36 (12.9)	0
Psychiatric disorders	3 (1.0)	0
Nervous system disorders	28 (10.0)	0
Eye disorders	15 (5.4)	0
Cardiac disorders	8 (2.9)	0
Vascular disorders	5 (1.8)	0
Respiratory, thoracic, and mediastinal disorders	34 (5.7)	1 (0.4)
Gastrointestinal disorders	120 (43.0)	6 (2.2)
Hepatobiliary disorders	8 (2.9)	0
Skin and subcutaneous tissue disorders	60 (21.5)	2 (0.7)
Musculoskeletal and connective tissue disorders	26 (5.7)	0
Renal and urinary disorders	17 (6.1)	0
General disorders and administration site conditions	102 (36.6)	5 (1.8)
Diagnostic exams	70 (25.1)	2 (0.7)

## 4 Discussion

To our knowledge, PEMBROREAL is the first real-world study reported in literature that focused on the use of pembrolizumab as a first-line treatment of NSCLC combined with pemetrexed and platinum in patients without driver mutation of *EGFR* and *ALK* and PD-L1 expression < 50%. The first consideration coming from the observation of the distribution of demographic and prognostic characteristics of patients included in the PEMBROREAL study is that the population normally treated in current clinical practice had a distribution of these characteristics significantly different from that enrolled in the clinical trial Keynote-189 (21, 24). As discussed in the Introduction in the real-world setting, we included patients with ECOG PS 2 usually excluded from clinical trials, patients with brain metastasis, and independently of their life expectancy. Another important difference regarding the population enrolled was the absence of patients with PD-L1 greater than or equal to 50% because of the limitation imposed by the Italian Medicinal Agency about reimbursability of the combination for these patients whom pembrolizumab was available as monotherapy. Moreover, we can observe some other differences, for example, relative to sex balance between Keynote-189 and PEMBROREAL. As can be seen in **Table 1**, 67.0% of the *PEMBROREAL* study participants were men and 33.0% were women. If compared with Keynote-189, the female sex usually has a more favorable prognosis (25) and is more represented in the pivotal clinical trial (38%) than in the real-life setting. Another relevant difference is the rate of patients with PD-L1 TPS <1% (44.1%) treated in the real-world setting compared to patients enrolled in Keynote-189, which included only 31% of patients with PD-L1 < 1% (21, 24). Considering only patients with PD-L1 <50% treated with pembrolizumab combined with pemetrexed and platinum, this percentage rose to 49.8%.

The percentage of patients with ECOG PS 0 was lower in the PEMBROREAL study (39.5%) compared to patients treated with pembrolizumab in the Keynote-189 study (45,5%). These two variables, PD-L1 expression and ECOG PS, are particularly relevant if we consider that PD-L1 end ECOG PS emerged as important prognostic factors from the univariate analysis conducted by our study team and from brief literature research (26–28). Between the pull of variables analyzed, smoker status seems to be the most relevant difference between patients treated in current clinical practice and in clinical trials. Never-smoker patients recruited in the Keynote-189 trial were only 11.2% versus 23.4% in our real-world study, but we have to consider that smoker status was unknown for 138 (49.5%) of the 279 patients included in the study. Nevertheless, this variable is very interesting considering the known differences in terms of response to immunotherapy documented in scientific literature (29–33).

The median RwPFS was 8.0 months, and this is consistent with findings from Keynote-189 in which PFS was 9.0 months in patients treated with pembrolizumab administered with pemetrexed and platinum. However, as previously stated, the results reached in the pivotal clinical trial were referred to as the intention-to-treat population, which also included patients with PD-L1 ≥ 50%, excluded from the *PEMBROREAL* study.

If we compare the RwPFS considering PD-L1 expression between the real-world setting and the clinical trial setting, PFS in the subgroup of patients with PD-L1 < 1% and with PD-L1 ≥ 1% and < 50% was very similar (5.8 months versus 6.2 and 9.4 months versus 9.9 months, respectively).

Despite the follow-up of 19.7 months, we did not reach the RwOS for patients included in our study. However, the survival rate was consistent with the findings from the *Keynote-189* trial. A 12-month survival rate in the intention-to-treat population of Keynote-189 was 69.8%, not so different if compared with the survival rate obtained in PEMBROREAL (66.1%). A greater difference could be observed if we consider the 24-month survival rate from Keynote-189 (45.7%) compared with PEMBROREAL (52.5%). This result seems to be better for patients treated in current clinical practice. Furthermore, if we consider the survival rate separately in the subgroup of patients with PD-L1 <1% and those with PD-L1 ≥ 1 and <50%, survival rate was very close between the pivotal clinical trial and the real-world study. Twelve-month and 24-month survival rates were 71.1% and 63.4% in Keynote-189 versus 71.8% and 59.4% in the PEMBROREAL study for patients with PD-L1 ≥ 1 or <50%, respectively, while for patients with PD-L1 < 1%, survival rates were 63.4% and 39.3% versus 59.0% and 43.2%.

From the analysis conducted on subgroups of interest, the PD-L1 expression and ECOG PS emerged as important prognostic factors influencing significant outcomes such as PFS and OS. Particularly remarkable were the PFS and OS results of patients with ECOG PS 2 (4.6 months) excluded from Keynote-189. This result is comparable with the PFS obtained for patients with ECOG PS 0–1 enrolled in the control arm of Keynote-189 (PFS: 4.9 months) and in the registrative study of pemetrexed combined with platinum as first-line treatment of NSCLC (PFS: 4.8 months) (21, 34) that did not include patients with ECOG PS 2. OS in the same subgroup as well was comparable to OS results from Keynote-189 in patients with better ECOG PS treated in the control arm (12.0 months vs. 10.8 months) and in patients treated in the registrative study of pemetrexed combined with platinum in the first-line treatment of NSCLC (OS 10.3 months). This is relevant to underscore because this result suggests that adding pembrolizumab to standard platinum-based chemotherapy made it possible to achieve the same results of chemotherapy alone for patients with ECOG PS 2 that generally were the subgroup with the worst prognosis normally excluded from recruitment in clinical trials. However, the limit of these considerations about this subgroup of interest included in the PEMBROREAL study is the underrepresentation of patients with ECOG PS 2 (*n* = 16, 5.9%); thus, this result should be interpreted with caution. Nevertheless, the value of poor ECOG PS as a prognostic factor for patients treated with immunotherapy is well explored in literature (35, 36), and our finding is consistent with what was reported about this subpopulation. The worst prognosis for these patients could be related to disease burden and comorbidities. Moreover, patients with poor ECOG PS are more likely to experience illness and mortality caused by the toxicity related to the immunotherapeutic regimen with three drugs combined and are less likely to receive further line of therapy aimed to prolong survival in case of treatment failure.

Response to treatment was very interesting and consistent with results from clinical trials. However, these results could be biased from the method of progression assessment as explained in Methods and Materials. Nevertheless, if we consider the control rate from Keynote-189 (84.6%), it was not so different from the value of the DCR estimated in PEMBROREAL (79.2%).

The results shown above seem to be very close to that obtained in Keynote-189. This finding could be unexpected if we consider that populations treated in the clinical trial cohorts are typically better selected by stringent inclusion and exclusion criteria and are generally healthier compared with patients treated in clinical practice (37). However, several factors can contribute to the overestimation of clinical outcomes in the real-world setting. First of all, the institutions selected to participate in our study are strongly oriented to cancer treatment. As stated previously, 13 out of the 16 involved institutions had a recognized role in cancer research. Additionally, PFS is generally overestimated in real-world studies. This issue may have been exacerbated by the COVID-19 pandemic contest, which could have resulted in fewer hospital visits (38).

Moreover, progression had to be determined radiologically as per RECIST criteria in Keynote-189 and was subject to blinded independent central review. On the other hand, patients in PEMBROREAL could have progression determined based on either radiological or clinical evidence. Future analyses to investigate the impact of this limitation on PFS would be of interest. OS is also generally overestimated in real-world studies. In terms of the effect of patients lost to follow-up because the reason for the loss “may” be related to the underlying patient health status. However, to avoid this confounding factor, we decided to cap the survival of patients lost at follow-up to the last contact documented in clinical records.

Regarding safety, findings from the PEMBROREAL study reveal that pembrolizumab associated with chemotherapy was well tolerated. If we compared AE incidence in patients enrolled in the experimental arm of Keynote-189 (99.8%) to the incidence reported in the real-world setting (44.4%), we could see that outside of the clinical trials in which all AEs are reported carefully in clinical records and a case report form, the incidences of AEs that physicians assess as important to report as meaningful are fewer than half. Gastrointestinal and blood and lymphatic system disorders are the most reported AEs, which is in accordance with findings from clinical trials but probably were related to the well-known effects of chemotherapy rather than pembrolizumab (39–41). Grade 3 and 4 AEs were rare in patients treated in current clinical practice (27 events). Most of the AEs that occurred did not require specific treatment of the event, and the others were easily managed by treating physicians pharmacologically or with dose reduction or temporary suspension, after which the patient was considered fit to restart treatment. Most concerns regarding treatment safety are related to adverse reactions going unreported, especially if they are mild or not clinically relevant.

The findings from the PEMBROREAL study confirm that pembrolizumab, when used in combination with pemetrexed and platinum salts, is effective and safe in treating metastatic non-squamous NSCLC in patients with PD-L1 < 50%. It is especially important to keep in mind the restrictions on reimbursement for the treatment discussed earlier. These limitations are based on a post-hoc analysis of the Keynote-189 trial, which was conducted with a sample size that was not adequate to determine the impact of the treatment on PFS and OS in this subgroup analysis. PFS and OS outcomes were broadly consistent with the Keynote-189 trial despite limitations associated with assessing disease progression in the context of a retrospective observational study that could cause an overestimation of RwPFS. The treatment was generally well tolerated with only a few cases of definitive treatment interruption due to toxicity, but perhaps also due to the general absence of a recognized standard second-line treatment for NSCLC patients without driver mutation previously treated with chemotherapy and immunotherapy (41–43). This fact could be the reason behind physicians not giving up on treatment toxicity but rather persevering until the toxicity becomes unmanageable (44).

## Data availability statement

The raw data supporting the conclusions of this article will be made available by the authors, without undue reservation.

## Ethics statement

The studies involving humans were approved by Ethics Committee of Vast Area of Romagna. The studies were conducted in accordance with the local legislation and institutional requirements. The participants provided their written informed consent to participate in this study.

## Author contributions

AC: Writing – original draft, Supervision, Project administration, Methodology, Investigation, Formal analysis, Data curation, Conceptualization. FF: Writing – review & editing, Visualization, Software, Methodology, Formal analysis, Data curation. ON: Writing – review & editing, Supervision, Methodology, Conceptualization. MCh: Funding acquisition, Writing – review & editing, Supervision, Software, Resources. MCo: Writing – review & editing, Supervision, Investigation, Conceptualization. PB: Writing – review & editing, Supervision, Conceptualization. SO: Writing – review & editing, Investigation. FE: Writing – review & editing, Supervision, Investigation, Conceptualization. VL: Writing – review & editing, Supervision, Investigation, Conceptualization. RL: Writing – review & editing, Supervision, Investigation, Conceptualization. PN: Writing – review & editing, Supervision, Investigation, Conceptualization. PM: Writing – review & editing, Supervision, Investigation, Conceptualization. FG: Writing – review & editing, Supervision, Investigation, Conceptualization. AG: Writing – review & editing, Supervision, Investigation, Conceptualization. GC: Writing – review & editing, Supervision, Investigation, Conceptualization. RP: Writing – review & editing, Supervision, Investigation, Conceptualization. PC: Writing – review & editing, Supervision, Investigation, Conceptualization. AP: Writing – review & editing, Supervision, Investigation, Conceptualization. SV: Writing – review & editing, Supervision, Investigation, Conceptualization. MV: Writing – review & editing, Investigation. ADF: Writing – review & editing, Supervision, Conceptualization. LC: Writing – review & editing, Visualization, Validation, Supervision, Project administration, Conceptualization. AD: Writing – review & editing, Visualization, Validation, Supervision, Project administration, Conceptualization. CM: Funding acquisition, Writing – review & editing, Supervision, Software, Resources, Project administration, Conceptualization.
