# Supplementary material for: A real-world retrospective, observational study of first-line pembrolizumab plus chemotherapy for metastatic non-squamous non-small cell lung cancer with PD-L1 tumor proportion score < 50% (PEMBROREAL)
Source: Front Oncol. 2024 Mar 27;14:1351995. doi: 10.3389/fonc.2024.1351995 (PMC11004281; doi:10.3389/fonc.2024.1351995)
Supplement: Supplementary file 1 [file DataSheet_1.docx]

**Supplementary Data**

**Supplementary Table 1:** Differences between eligibility criteria in Keynote189 and eligibility criteria established for National Health system reimbursement

| Keynote – 189 – key patients elegibility criteria | Elegibility Features for patients treated in clinical practice |
| --- | --- |
| NSCLC stage IV | NSCLC stage IV |
| EGFR wild type | EGFR wild type |
| ALK wild type | ALK wild type |
| Untreated metastatic adenocarcinoma | Untreated metastatic adenocarcinoma |
| ECOG PS 0-1 | ECOG PS 0-2 |
| Aspettativa di vita superiore ai 3 mesi | Not specified life expectancy |
| Whatever is TPS of PD-L1 | TPS di PDL-1< 50% |
| No active brain metastasis | Indipendently by the presence of active brain metastasis |

**Supplementary Table 2:** Reasons for Pembrolizumab Treatment discontinuation

| **Reason** |  |  | **Overall Patients**  **n=279** |
| --- | --- | --- | --- |
| Progressive disease or clinical progressive disease | | | 154 (55.2) |
| Unacceptable toxicity |  |  | 15 (5.4) |
| Lost to follow up |  |  | 22 (7.9) |
| Patient’s decision |  |  | 0 (0.0) |
| Clinical decision |  |  | 9 (3.2) |
| Death |  |  | 21 (7.5) |
| Not specified |  |  | 0 (0.0) |
| Therapy is still ongoing at the cutoff data |  |  | 58 (20.8) |

**Supplementary Table 3:** Response Rate

| **Best overall response** | **Overall patients**  **n=279** |
| --- | --- |
| Complete response | 5 (1.8) |
| Partial response | 53 (19.0) |
| Stable disease | 62 (22.2) |
| Progressive disease | 54 (19.4) |
| Not yet documented | 105 (37.6) |

**Supplementary Table 4**: Adverse reaction management

| **Action taken** | **Overall patients**  **n=279 (%)** |
| --- | --- |
| Suspension of one or more drug | 25 (8.96) |
|  |  |
| Permanent discontinuation of the treatment | 12 (4.30) |
|  |  |
| Dose reduction of one or more drugs | 29 (10.39) |
|  |  |
| Specific pharmacologic treatment for toxicity management. | 65 (23.3) |
|  |  |
| Hospitalization or prolongation of hospitalization | 16 (5.73) |
|  |  |

**Supplementary Table 5**: Univariables and multivariable model for PFS

| **Variables** | **Univariable models** | | | **Multivariable model** | | |
| --- | --- | --- | --- | --- | --- | --- |
|  | **Hazard Ratio** | **p value** | **95% CI** | **Hazard Ratio** | **p-value** | **95% CI** |
| PDL1 1-49% vs PDL1 <1% | 0.69 | 0.010 | 0.52-0.92 | 0.69 | 0.011 | 0.52-0.92 |
| PS ECOG 1-2 vs PS ECOG 0 (main effect) | 4.02 | <0.001 | 1.88-8.60 | 3.97 | <0.001 | 1.86-8.48 |
| PS ECOG 1-2 vs PS ECOG 0 (time dependent effect) | 0.56 | 0.005 | 0.37-0.84 | 0.56 | 0.005 | 0.38-0.82 |

**Supplementary Table 6**: Univariables and multivariable model for OS

| **Variables** | **Univariable models** | | | **Multivariable model** | | |
| --- | --- | --- | --- | --- | --- | --- |
|  | **HR** | **p-value** | **95% CI** | **HR** | **p-value** | **95% CI** |
| PDL1 1-49% vs PDL1 <1% | 0.62 | 0.009 | 0.43-0.90 | 0.61 | 0.008 | 0.42-0.88 |
| PS ECOG 1-2 vs PS ECOG 0 | 1.63 | 0.016 | 1.09-2.43 | 1.64 | 0.015 | 1.10-2.44 |

**Supplementary Figure 1:** PFS in subgroup of interest. A) Kaplan – Meier distribution of real world PFS of patients with PD-L1 level greater or equal to 1 but lower than 50 versus patient with PD-L1 < 1%. B) Kaplan – Meier distribution of real world PFS of patients with ECOG PS 0 versus ECOG PS 1 versus ECOG PS 2

**
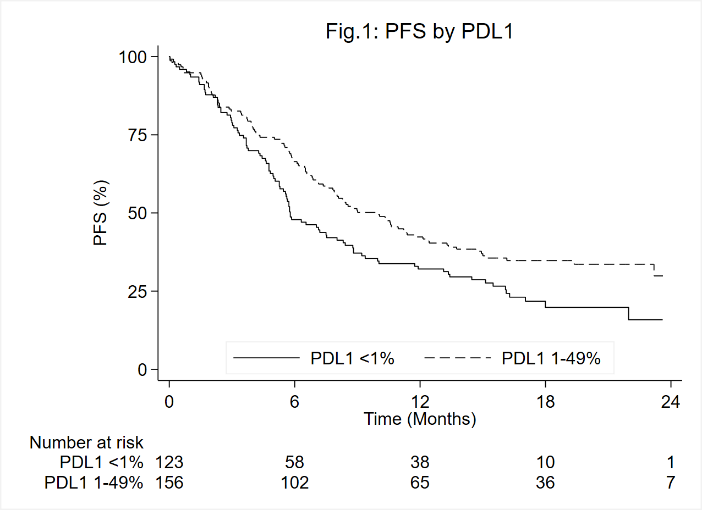

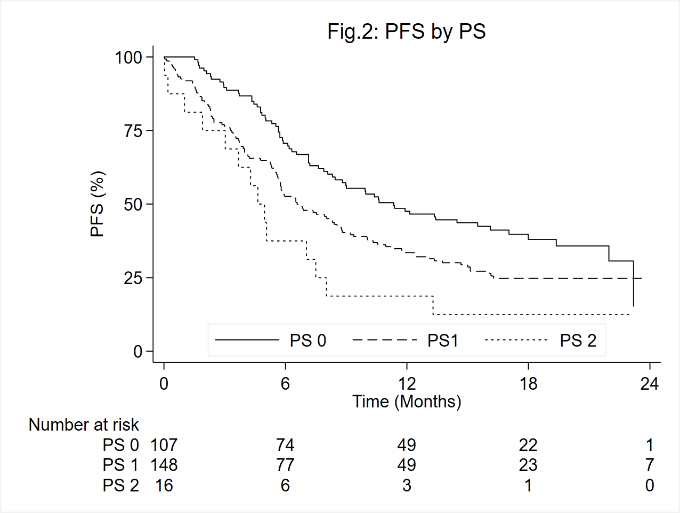
**

B

A

**Supplementary Figure 2:** OS in subgroup of interest. A) Kaplan – Meier distribution of real world OS of patients with PD-L1 level greater or equal to 1 but lower than 50 versus patient with PD-L1 < 1%. B) Kaplan – Meier distribution of real world OS of patients with ECOG PS 0 versus ECOG PS 1 versus ECOG PS 2 C) Kaplan – Meier distribution of real world OS male patients versus female patients.

**
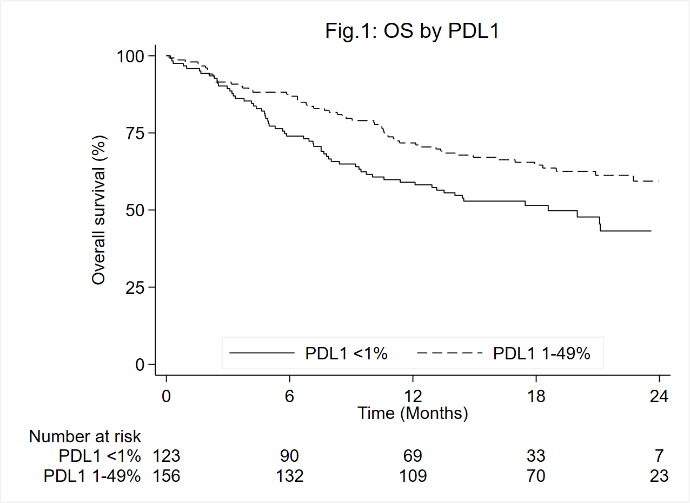

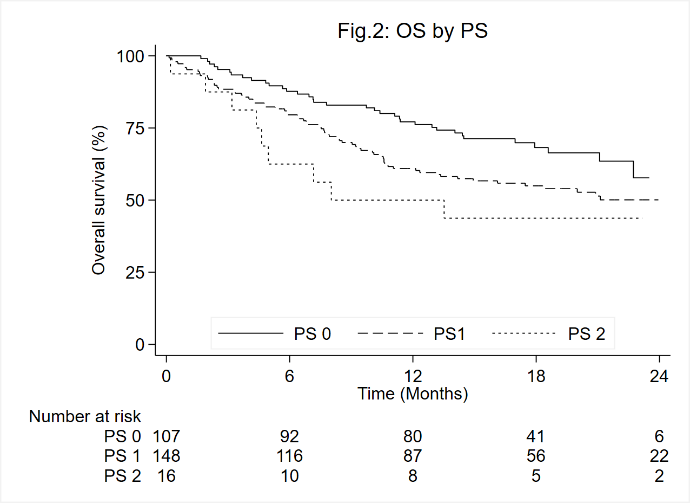
**

B

A

**
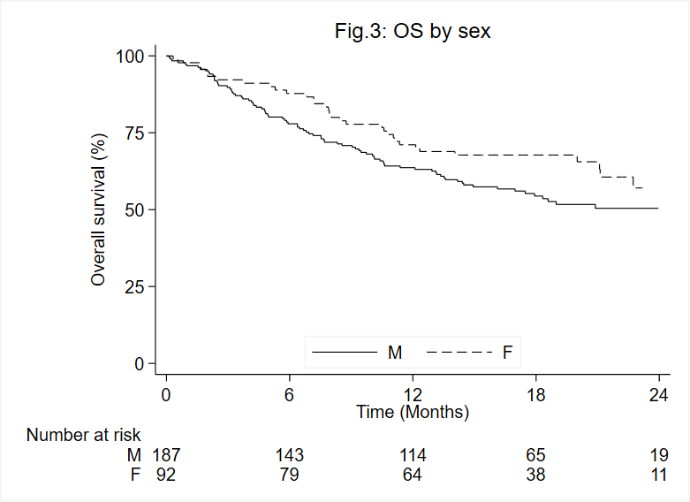
**

C
